# Research on the Accelerated Fatigue Experiment Method of the Crankshaft Based on a Modified Particle Filtering Algorithm and the Fatigue Crack Growth Property

**DOI:** 10.3390/ma19030481

**Published:** 2026-01-25

**Authors:** Jiahong Fu, Songsong Sun, Xiaolin Gong, Shanshan Shen, Nana Jiang, Jianmin Juan

**Affiliations:** 1 College of Engineering, Hangzhou City University, Hangzhou 310015, China; fujh@zucc.edu.cn; 2 College of Automobile and Traffic Engineering, Nanjing Forestry University, Nanjing 210037, China; 3 College of Mechanical and Electrical Engineering, Zhejiang Industry Polytechnic College, Shaoxing 312000, China; 4 Zhejiang Times Auto Sparts Parts Co., Ltd., Jiaxing 314400, China

**Keywords:** crankshaft, bending fatigue, remaining life prediction, statistical analysis

## Abstract

Crankshafts are among the most important parts of modern internal combustion engines. Owing to the power transmission demand, sufficiently high strength is usually necessary for the application of the component. In this paper, a new crankshaft bending experimental method was proposed to shorten the corresponding test. A modified particle filtering algorithm approach was proposed for predicting the remaining fatigue life of a crankshaft during bending fatigue experiments. The predicted fatigue life was used to replace the actual experimental results for further analysis if the accuracy requirements were fulfilled; in this way, the experimental duration was obviously shortened. The main conclusion drawn from the research is that, compared with the traditional particle filtering algorithm approach, the modified particle algorithm approach proposed in this paper can more accurately predict the remaining fatigue life of a crankshaft using less experimental data, which makes it possible to circumvent actual bending fatigue experiments of crankshafts in providing theoretical guidance for the design process.

## 1. Introduction

Currently, diesel engines are widely applied in actual engineering applications. Compared with gasoline engines, diesel engines can usually provide much more power and energy [1,2]. As a result, more critical demands are required for the fatigue strength of key parts, such as the crankshaft, to ensure the necessary safety and reliability [3,4].

With respect to this topic, innovative work has emerged in recent years. Hernandez studied the fatigue failure mechanism of the selected crankshaft and has found out three primary causes, namely inadequate heat treatment, problems in material selection and insufficient bushing adjustment which caused heating and surficial deformation, hence causing the fretting-fatigue of the component [5]. Hosseini employed acoustic emission entropy technique with the help of which the subject of fatigue cracks of a crankshaft was studied; therefore, the scope of data was significantly decreased, and the latter method was far more feasible and affordable for real-time crankshaft health monitoring [6]. Yanping Wang performed an analysis on a fractured 42CrMo crankshaft of a heavy truck and stated that a crack originated in the intersection of threaded bottom hole column surface and taper surface. The key causal factor of the fracture consisted of the stress concentration due to the absence of an apparent transition fillet in the same location, and the transformation of the metallographic structure of surface layer of the bottom hole under the high machining temperature [7]. Shuailun Zhu came up with the failure analysis and numerical simulation of a particular crankshaft in association with the corresponding answer surface optimization; in this manner, the stress and deformation of the part was explicitly assuaged [8]. Leitner evaluated the strength of the electroslag remelted 50CrMo4 steel crankshafts based on the concept of multi-axial fatigue and established a better model, which can predict the fatigue strength of a particular component as compared to earlier models that used empirical investigation [9]. The outcome of the fatigue test and 2D-FE examination of the J-integral was combined by Ahmed, and this test demonstrated the overall change in the fracture characteristics [10]. Qin used the critical plane technique to scrutinize the effect of residual stress field on the property of fatigue, and the suggested model could match the requirement of accuracy [11].

Bending fatigue is the widespread type of the crankshaft damage at present. As such, a relative fatigue strength analysis will also be required to offer cursory theoretical direction to the expenditure of the components [12]. This work is typically performed through standard resonant bending fatigue experiments in actual automobile engineering. Such a test is carried out with professional machines and is capable of imitating the loading state of the crankshaft, by offering alternating bending force. Nevertheless, the experiment phase can be a bit prolonged (in some cases, it takes months) due to the fact of excessive cycle fatigue [13,14]. Moreover, the theory of fatigue reliability suggests that the phenomenon of obvious dispersion may likely be identified from the data of fatigue test. Because of it, a bigger experimental sample size is required in statistical analysis, and more time and expenses are required. Therefore, establishing the crankshaft fatigue characteristics in a relatively small duration of the experiment can result in a significant breakthrough in the usage of the part [15].

Conversely, the most important parameter in the fatigue test process is the fatigue life in the failure load cycle. When the behavior of failure can be predicted, the remaining part of experiment can be omitted to conserve the time and cost. Currently this remaining life prediction research has been extensively used in batteries and bearings. Nevertheless, less literature has been published in the prediction methods of leftover fatigue life in crankshafts. In the former work, we used the classical method of a particle filtering algorithm to find the remaining fatigue life of a crankshaft. The conclusions were that this approach can give credible projections in most of the cases, but in some instances, there is the occurrence of obvious errors (more than 15%). Moreover, time saving is not apparent (around 10% of the entire experimentation procedure) [16,17]. Also, some other related methods such as the Kalman filtering algorithm were selected in analyzing the fatigue property of the crankshaft; the results showed that the accuracy sometimes contained obvious errors, which was not suitable for applications [18,19,20]. In addition, the selection of the damage failure parameter also affects the accuracy of the predictions [19,20].

Generally speaking, although some algorithms have already been adopted in predicting the residual fatigue life of the crankshaft, but the accuracy of the predictions cannot be guaranteed in some conditions. The main reason for this phenomenon is that these studies were conducted based on the analysis of the data recorded within the partial period. The fatigue damage accumulation property throughout the whole process has rarely been studied. In addition, during these studies, the values of the main model parameters were set as default, which may not be suitable for the application scenarios. In addition, although more training data can improve the accuracy of prediction, the timesaving effect is also weakened, which decreases the promotional value. Thus, how to determine the remaining fatigue life of the crankshaft during its experiment process as early as possible is still a problem.

To overcome this problem, this paper suggests a better particle filtering algorithm to estimate the remaining fatigue life of a crankshaft. To identify the relationship between the fatigue life and the fatigue crack length, firstly, it was established basing on the suggested finite element method and dynamic response of the part. Secondly, there was an upgrading of a particle filtering algorithm, according to new model parameter optimization, in forecasting the remaining fatigue life of the crankshaft throughout the bending fatigue testing. Lastly, the results that were predicted to have good accuracy were selected to substitute the practical results in the evaluation of the fatigue strength of the crankshaft. The overall result of the above work is the fact that the proposed method could offer almost the same results as the original experimental data in a shorter time span of the experiment, thus being useful in future extension to the actual engineering work.

## 2. Method

### 2.1. Experimental Equipment

**Figure 1:**
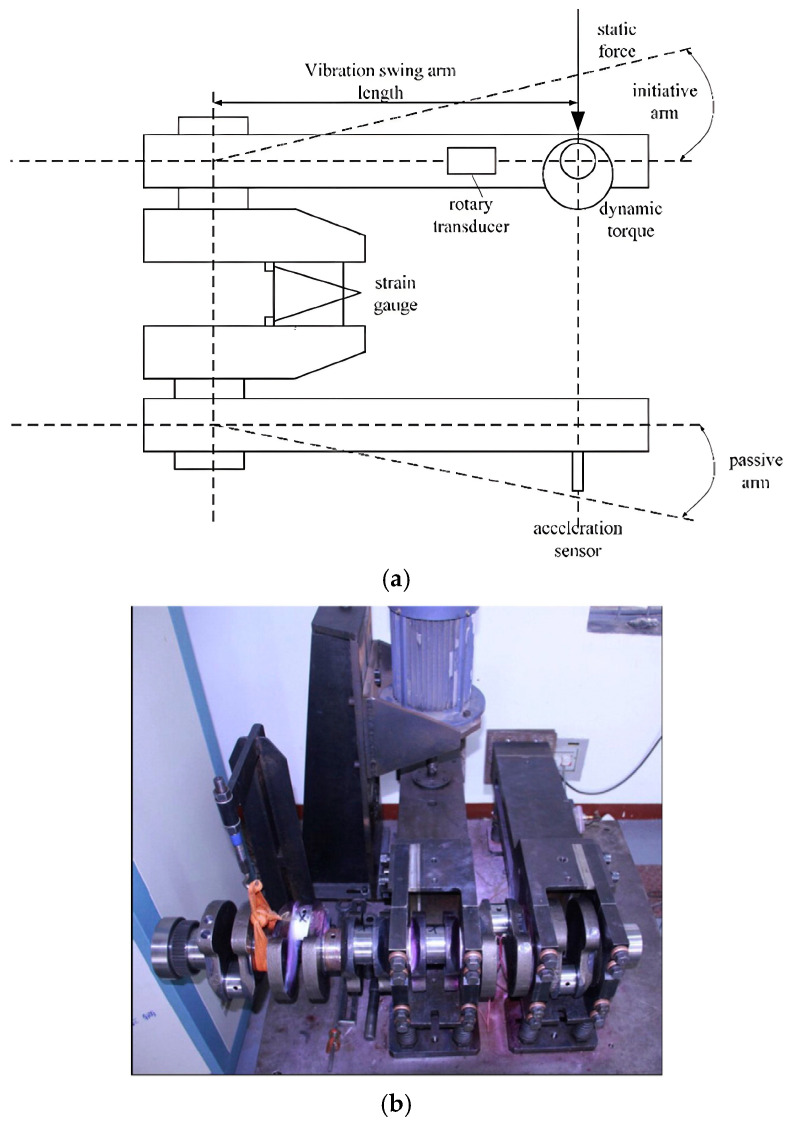
Bending fatigue test equipment for the crankshaft: (**a**) the structure diagram of the equipment [17]; (**b**) the real photo of the equipment [19].

In this work, the research object is the bending fatigue of a crankshaft. At present, this property is usually determined through professional bending fatigue tests. Figure 1 shows the detailed components of the equipment, from which it can be found that the crankpin of the crankshaft is tightly clamped by two arms. During the experimental process, the rotating motor continuously applies an alternating impact load on the master arm. Owing to resonance, a responsive alternating bending moment is applied on the crankpin. This results in the accumulation of fatigue damage in the stress concentration area, as well as a reduction in the stiffness of the whole system. When the degree of fatigue damage to the crankpin has reached a certain value, the crankpin is considered broken [21,22]. The serial number of the occupational standard applied in this experiment is QC-T637-2000 [23].

In this paper, the material of the crankshaft is 42CrMo, a typical kind of high-strength alloy steel. Table 1 shows the detailed information of the material. In addition, the tensile strength of the material is 874 MPa, and the corresponding yield strength is about 667 MPa.

During the experiment process, the temperature is just the room temperature (about 25 °C). Based on this equipment, corresponding bending fatigue experiment of the crankshaft can be conducted.

### 2.2. The Statistical Analysis Method

**Figure 2:**
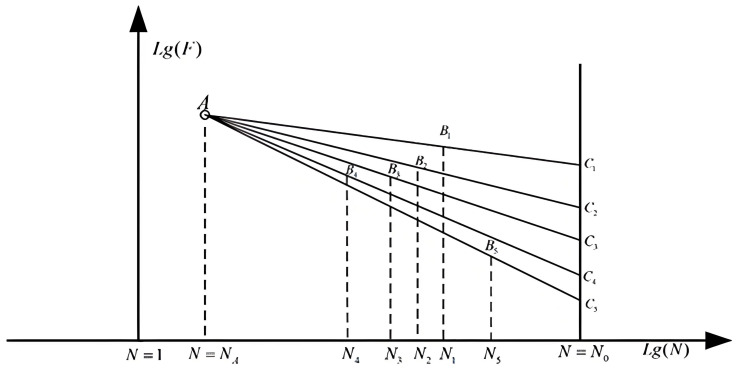
The load-life relationship according to the SAFL (statistical analysis for fatigue limit) method [19].

According to the theory of fatigue and reliability, fatigue test results usually contain obvious data dispersion property. Sometimes, the relative difference between the values of the two groups of fatigue test can be as much as twice or more. This makes suitable design and corresponding statistical analysis necessary. At present, the most commonly used method in this field is the SAFL approach. Figure 2 shows the theoretical basis of this method, in which it can be found that in each case, one specimen was tested under the given stress level. The distribution of the fatigue limit load is determined by the mapping approach.

$F_{A}$ is the load amplitude which can be determined through a least squares fit approach of the experiment data.

### 2.3. The Remaining Fatigue Life Prediction Method

As described above, during the bending fatigue experiment for the crankshaft, an alternating bending moment is applied to the sample until it breaks. At present, this process usually lasts days or weeks, which may result in high costs. A possible approach to shorten this process is to accurately predict the fatigue failure time node before actual failure occurs. At present, various kinds of remaining life prediction methods have been widely applied in actual engineering, among which the particle filtering algorithm is considered to be an effective choice [16]. This method is a statistical filtering method based on Monte Carlo theory and recursive Bayesian estimation, which applies Monte Carlo theory in the integral computation of recursive Bayesian estimation according to the law of large numbers. The theoretical basis of this approach can be expressed in the following steps.

Step 1: In this step, the state vector $x_{k}$ of the system is expressed as

$$x_{k}=f\left(x_{k-1},w_{k-1}\right)$$

where $x_{k-1}$ is the input vector at the (*k* − 1)th step and where $w_{k-1}$ is the sampling noise of the particle at the same step. In addition, the sampling noise is distributed according to the Gaussian model, which can be expressed as

$$w_{k-1}∼(0,Q_{k-1})$$

where $Q_{k-1}$ is the covariance matrix of the process noise. The state transition equation of the system is defined as

$$x_{k}=x_{k-1}+a\sqrt{\left|x_{k-1}\right|}+w_{k-1}$$

where a is the state transition coefficient.

Step 2: In this step, the observation equation is built to obtain the observed value of the system via actual measurement. The observed value related to the state vector is defined as

$$z_{k}=h(x_{k},v_{k})$$

where $v_{k}$ is the noise vector of the observed value. Similar to the sampling noise, the noise of the observed value is also distributed according to the Gaussian model, which can be expressed as

$$v_{k}∼N\left(0,R_{k}\right)$$

where $R_{k}$ is the covariance matrix of the observed value noise.

Step 3: In this step, the initial particle set is created in the state space by randomly generating *N* initial particles ${\left\{x_{0}^{i}\right\}}_{i=1}^{N}$, among which the particle $x_{0}^{i}$ is an *N*-dimensional vector. In addition, the initial weighted values of all the particles are the same, which can be expressed as

$$w_{0}^{i}=\frac{1}{N}$$

where $w_{0}^{i}$ is the weighted value of particle $x_{0}^{i}$. On the basis of this and the state transition equation, the prediction of each particle value at the *kth* time step on the basis of the particle value obtained at the (*k* − 1)*th* time step can be expressed as

$$x_{k}^{i}=f\left(x_{k-1}^{i},w_{k-1}^{i}\right)$$

Step 4: On the basis of the observed value and the state transition equation, the weighted value of each particle can be calculated as

$$w_{k}^{i}=w_{k-1}^{i}p(z_{k}/x_{k}^{i})$$

where $p(z_{k}/x_{k}^{i})$ is the likelihood function, which is equal to the probability of observing $z_{k}$ under state $x_{k}^{i}$. On this basis, the weighted values of all the particles according to the normalization processing approach can be determined as

$$\hat{w_{k}^{i}}=\frac{w_{k}^{i}}{\sum_{j=1}^{N}w_{k}^{j}},i=1,2\ldots ,N$$

Step 5: In this final step, when the number of effective particles is lower than the specified threshold value, the resample work starts for further prediction, and the relationship can be expressed as

$$N_{eff}=\frac{1}{\sum_{i=1}^{N}{\left(\hat{w_{k}^{i}}\right)}^{2}}$$

$$\hat{l_{k}}=\sum_{i=1}^{N}\hat{w_{k}^{i}}y(x_{k}^{i})$$

where $N_{eff}$ is the number of effective particles and where $\hat{l_{k}}$ is the output of the prediction. With this approach, the prediction of the remaining fatigue life can be achieved [16].

According to a previous study [16], when the traditional particle filtering algorithm is applied for remaining fatigue life prediction, obvious errors can sometimes be found between the predicted data and the actual experimental data. The main reason for this phenomenon can be explained in two ways. First, during the iteration process, most of the particles are within the lower contribution area, which easily results in degeneration during the iteration cycle. Second, the values of the model parameters are generally steady, and fulfilling the demands of different application objects is difficult.

**Figure 3:**
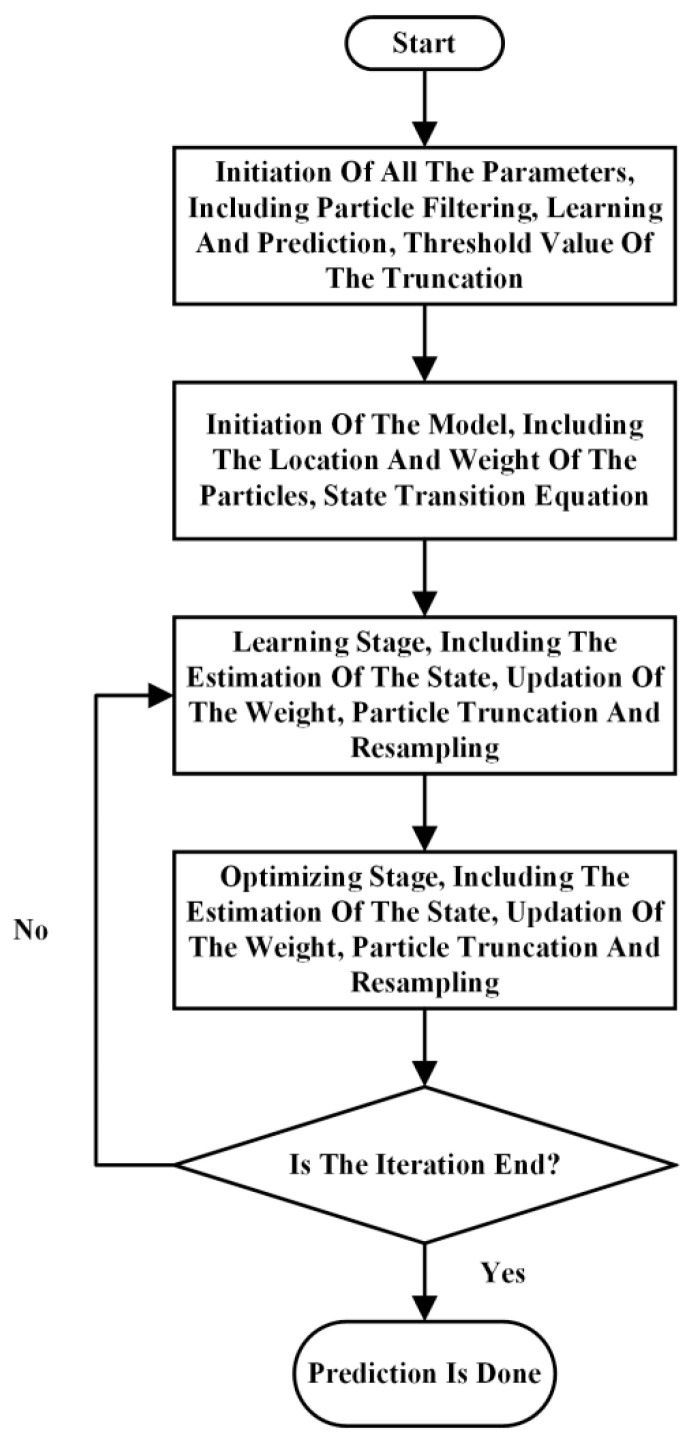
The detailed process of the modified particle filtering algorithm.

To solve this problem, a modified particle filtering algorithm method is proposed to improve the accuracy of prediction. The optimization work is conducted in two ways. First, a group of experimental data recorded throughout the whole fatigue failure process is chosen as the learning object model, during which the new state estimation and weighted value update are conducted. Second, a parameter modification stage is added according to the given equations. During the learning process, the state update equation is defined as

$$x_{k}=x_{k-1}+a{\left(\sqrt{\left|x_{k-1}\right|}\right)}^{m}+\sqrt{Q}\cdot \varepsilon_{t}^{i}$$

where $\varepsilon_{t}^{i}$ is Gaussian noise $N\left(0,1\right)$. For this modified state transition equation, $a{\left(\sqrt{\left|x_{k-1}\right|}\right)}^{m}$ is used to simulate the gradual process of evolution, and $\sqrt{Q}\cdot \varepsilon_{t}^{i}$ is an uncertain parameter. During the parameter modification stage, the model parameters are modified according to the observed error. The theory of this approach is expressed as follows

$$a=a+\eta \cdot error_{t}\cdot {\hat{x}}_{t}$$

$$Q=Q+c\cdot \left|error_{t}\right|$$

$$R=R+c\cdot \left|error_{t}\right|$$

where $error_{t}$ is the observed error at time node *t*, η is the learning rate and *c* is the relative correction factor for the covariance matrix. The other parameters are the same as those of the traditional particle filtering algorithm. The detailed process of this modified particle filtering method is shown in Figure 3. The whole prediction process was conducted based on the software Matlab (the version is 2012).

### 2.4. Fatigue Failure Parameter Criterion

On the basis of the modified particle filtering algorithm proposed above, it is possible to predict the remaining fatigue life of a crankshaft. On the other hand, during the fatigue experiment process, usually one or more failure criterion parameters are selected when evaluating the degree of damage accumulation. According to previous studies, the fatigue crack length is considered an effective choice in this situation. For this parameter, it is possible to determine the fatigue crack length indirectly via a combined finite element analysis and vibration method. The basic principle of this method is that during the fatigue experiment, the size of the fatigue crack affects the stiffness of the system. Thus, the size of the crack can be determined by measuring the dynamic response of the system [20]. The whole process can be divided into three steps in all.

Step 1: The initial finite element model was built to study the dynamic property of the crankshaft with on crack. The accuracy of the model was verified by corresponding dynamic test.

Step 2: The crankshaft has been tested according to the standard bending experiment to a certain load cycle. Then, the dynamic property of the broken crankshaft was recorded by the same dynamic test method above.

Step 3: The size of the fatigue crack was added to the finite element model to determine the dynamic property of the broken crankshaft at the same time node. When the simulation result is in accordance with the test result, the added fatigue crack size is just the actual fatigue crack size.

## 3. Results

### 3.1. Fatigue Crack Analysis Results

**Figure 4:**
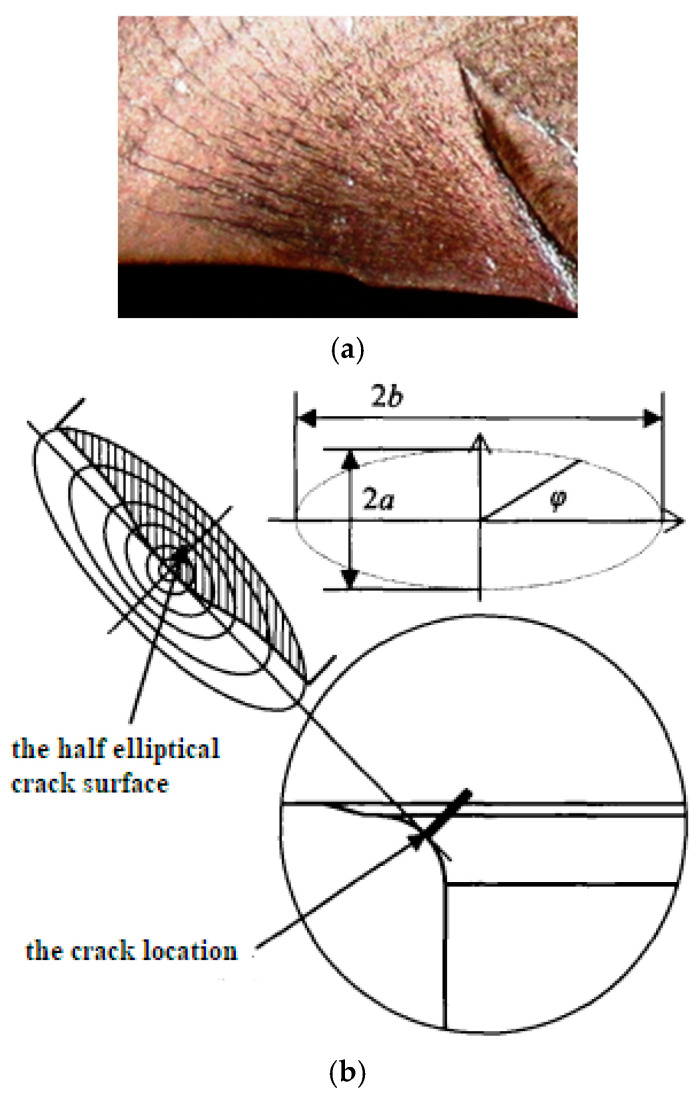
Fracture surface of a broken crankshaft: (**a**) the real photo of the fracture surface; (**b**) the structure diagram of the crack surface [19].

On the basis of the methods proposed above, it is possible to conduct research on the corresponding fatigue properties of crankshafts. In this work, the research object is a type of steel crankshaft from a six-cylinder diesel engine. Figure 4 shows the fracture surface of the broken sample, from which it can be found that the shape of the fatigue fracture surface is elliptical. In addition, typical fatigue bibbing lines can be observed around the crack initiation area because of the high degree of cycle fatigue.

**Figure 5:**
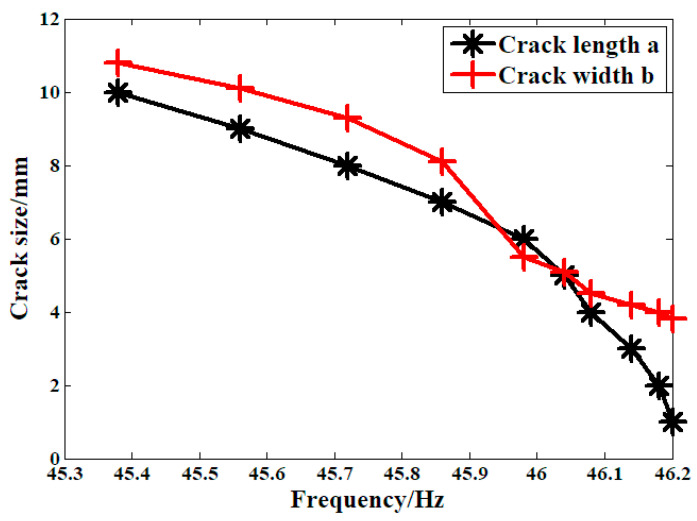
Relationship between fatigue crack size and dynamic system properties.

Figure 5 shows the relationship between crack size and the dynamic properties of the system, where a is the depth of the crack surface and b is the width of the crack surface. According to the experimental standard, when the decrease in the first-order system inherent frequency reaches 1 Hz, the sample is determined to be broken. For this crankshaft, when the crack depth reached zero, the crankshaft was considered to be broken.

### 3.2. Prediction Results

**Figure 6:**
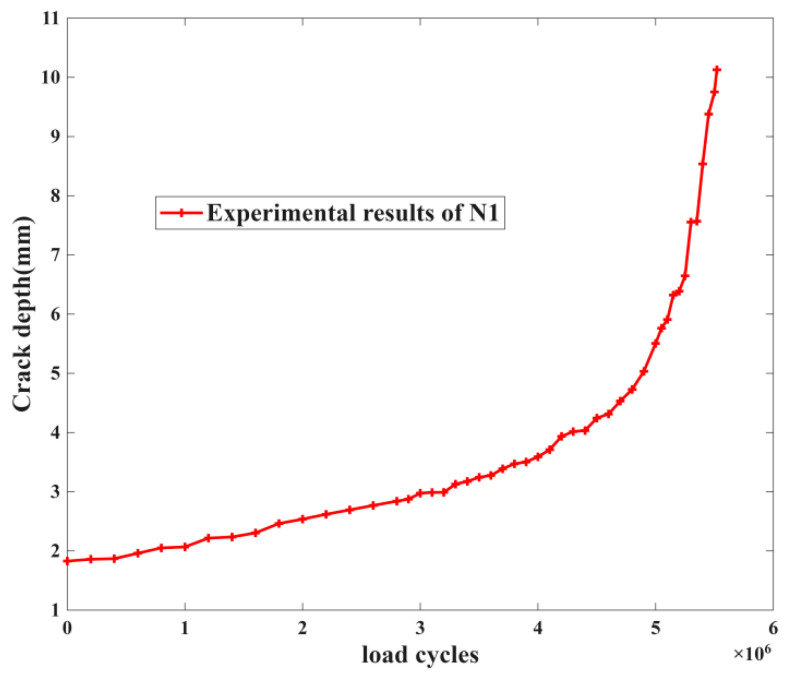
The fatigue crack growth process of crankshaft N1.

Based on the above analysis of the fatigue crack of the crankshaft, the remaining fatigue life can be predicted. In general, the remaining fatigue life is the number of load cycles between the given crack length and 10 mm. On the other hand, according to the method used in this study, before working out predictions, the model should be trained based on the evolution of the fatigue process over the entire period of fatigue failure. In Table 2, we have the results from the crankshaft experiment: it can be seen that the load cycles in four groups (two, three, four and seven) are larger, which may cause more costs. According to the above research method, the third group is selected as the training data, with the serial number of N1. The fatigue crack growth process of this crankshaft obtained by the above dynamic method is shown in Figure 6.

**Figure 7:**
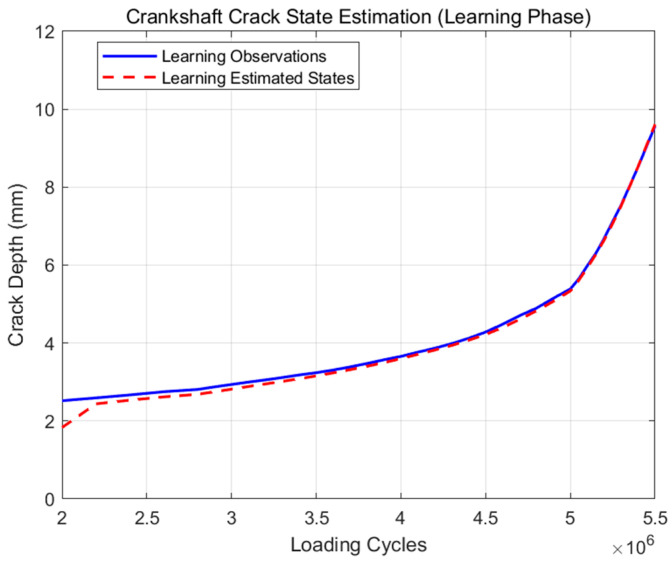
Learning results for crankshaft N1.

For the training data shown in Figure 6, it is obvious that when the crack length reaches 10 mm, the growth speed begins to increase rapidly. This is in accordance with the failure criterion of the standard. The fatigue life is approximately 5.52 × 10^6^. On the contrary, based on the fracture mechanics theory, the growth behavior of the fatigue cracks throughout the entire failure process is divided into three stages: the initial stage, the stable growth stage, and the accelerated growth stage. When comparing the values of each stage of fatigue crack growth speed, there is usually a noticeable difference. So in this study, the fatigue crack growth process from 1 mm to 2 mm which has been recorded as the train data, from 2 mm up to 10 mm, is the remaining fatigue life or the number of loading cycle for the next 8 mm, and the learning method which is proposed above would be used in building the modified particle filtering algorithm. Learning results are as shown in Figure 7.

As shown in Figure 7, at the beginning stage of the prediction, there are obvious errors between the predictions and the original experimental data. With time, the accuracy quickly improves. When the fatigue crack length reaches 5 mm, the prediction and original experimental data are mostly the same. Generally, after learning, the modified particle filtering model can accurately simulate the fatigue crack growth process of crankshaft N1.

**Figure 8:**
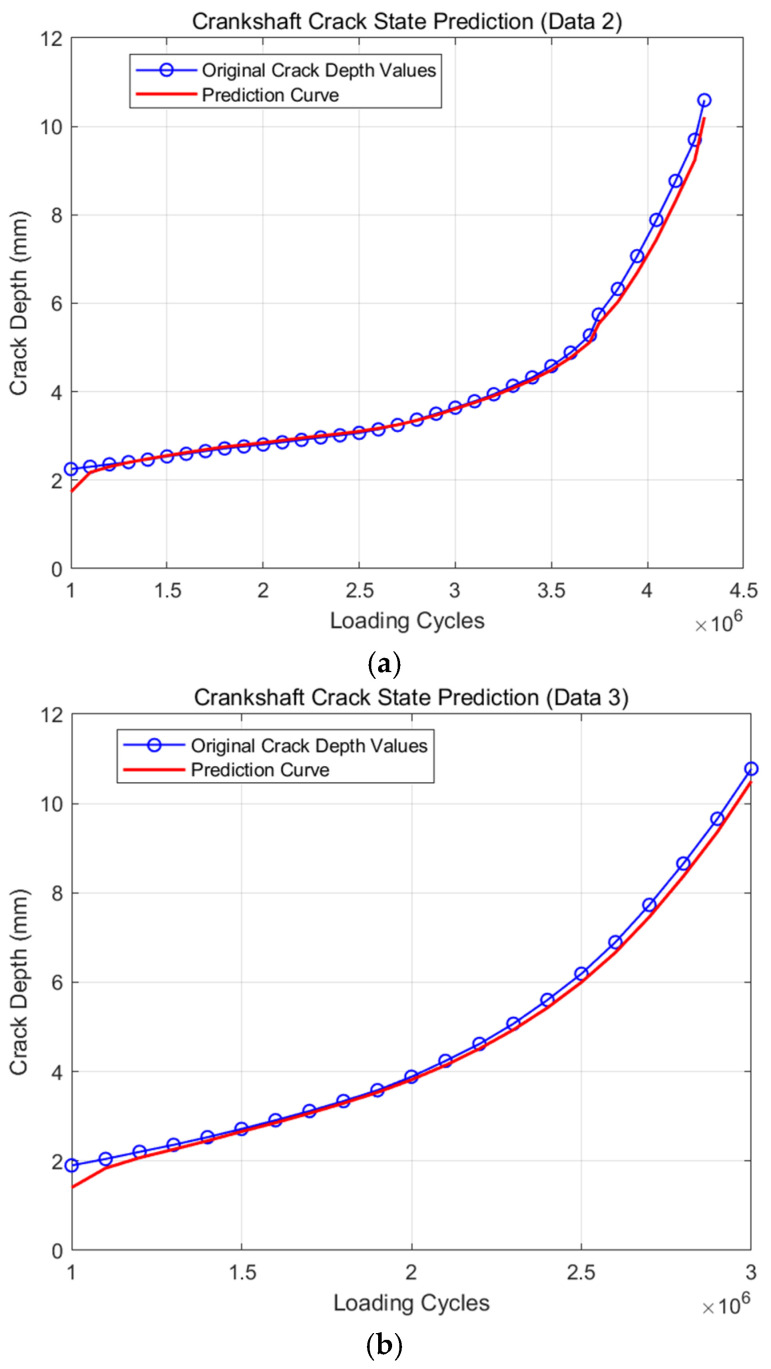

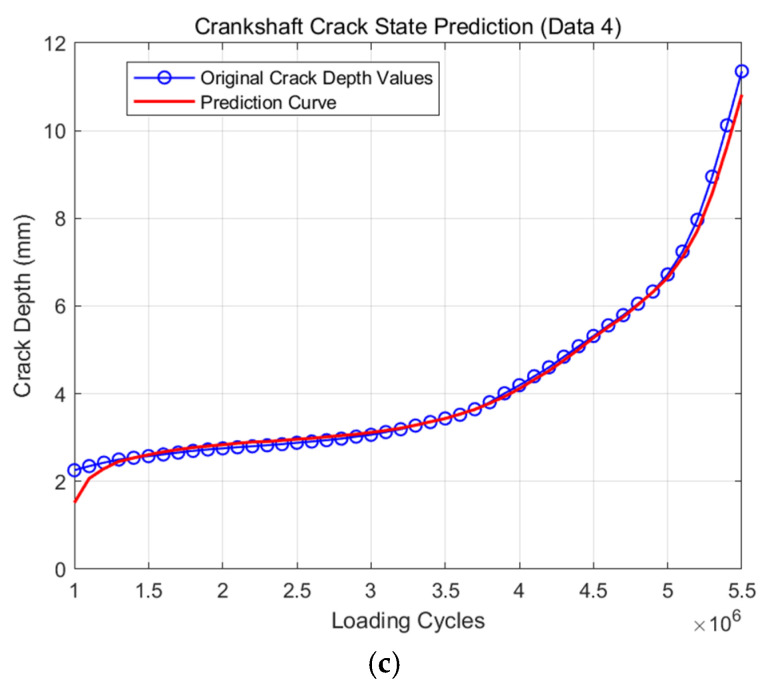
The prediction results of all the other three groups: (**a**) the prediction results of crankshaft N2; (**b**) the prediction results of crankshaft N3; (**c**) the prediction results of crankshaft N4.

The remaining fatigue life can also be predicted from any other groups of crankshaft bending fatigue experiments on the basis of a particular model. To further study the universality of the proposed model, take the other three groups of crankshafts for research (the second, the fourth and the seventh experiment results). The serial numbers of these three groups are N2, N3 and N4, respectively. Detailed results of prediction are shown in Figure 8.

From Figure 8a, it can be seen that when the modified model is used to predict the remaining fatigue life of crankshaft N2, the prediction results are very similar to the original experiment, with a relative error of less than 1.5% at each time node. As shown in Figure 8b, regarding the other two groups, the value of the error is also less than 2%. In general, this model can accurately predict the remaining fatigue life of more than a few groups of crankshafts during the crankshaft bending fatigue experiment.

Table 3 displayed the comparison of prediction by using traditional and modified particle filter. Compared with the traditional particle filtering algorithm, the prediction using the modified particle filtering method can not only achieve more accurate results but also reduce the experimental time. These two advantages make it worth promoting and using.

### 3.3. Statistical Analysis Results

**Figure 9:**
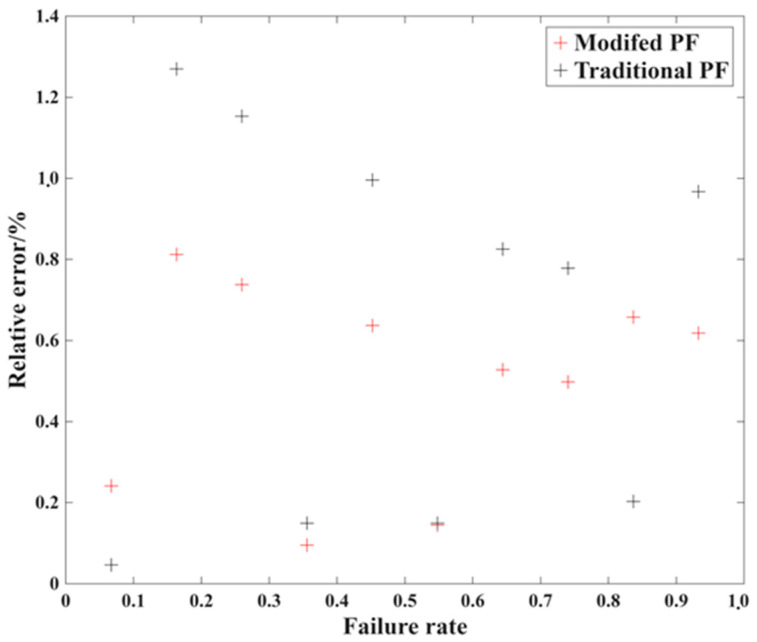
The relative error of the statistical analysis results based on different PF models.

From the prediction result above, the clear conclusion can be made: the modified particle filtering algorithm can predict the remaining fatigue life of crankshaft accurately and also shorten the experiment time to reduce the experimental cost. On the contrary, according to the actual engineering requirements, the most frequently employed method of assessing the fatigue property of the crankshaft is the fatigue limit load, which is mostly obtained via the SAFL (Statistical Analysis for Fatigue Limit) method: Figure 9 is the relative error of the fatigue limit load statistical analysis result with the original and prediction under different failure rate, and it can be seen that the relative error is in the range of 1%. Considering that there will also be certain deviations due to load calibration itself, this is more than sufficient.

### 3.4. Discussion

From the research above, it is clear that the proposed accelerated bending fatigue method for the crankshaft can effectively shorten the experiment period. In previous studies, some other methods such as the Kalman filtering algorithm are also selected in investigating this problem. The results showed that these methods could not avoid generating errors during the predictions. Otherwise, the training data should be more enough, which would result in the reduction of the timesaving effect. The main reason for this phenomenon is that during these studies, the values of the main model parameters are just set according to the default constant, which may not be suitable to be applied in this condition [16,17,18,19,20].

In this paper, the model was trained based on the fatigue damage parameter recorded throughout the whole fatigue damage process; in this way, the fatigue property of the part can be taken into consideration in advance. Thus, the accuracy has been improved obviously. In addition, the material of the crankshaft in this paper is 42CrMo. According to the theory of fracture mechanics, the fatigue crack growth speed of this kind of material is generally steady before the final fracture happens. Thus, the fatigue crack can be selected as the failure determination factor [24].

At present, remaining life prediction research has been widely carried out in recent years. However, the main research objects in these studies are the working life of the batteries or bearings [25,26]; the remaining fatigue life prediction research is relatively less reported in some degrees. In addition, among the remaining fatigue life predictions, some experts predicted the remaining fatigue life based on several sources of parameters such as stress or load [27]. In this paper, the prediction was just achieved based on the fatigue crack size parameters. For the resonant crankshaft bending fatigue test equipment widely applied today, the dynamic property of the crankshaft is recorded throughout the experiment process automatically; in this way, the fatigue crack size is also easy to determine. Secondly, for the other similar research, some other kinds of additional tests or experiments such as the SEM analysis or acoustic emission signal analysis should be provided for assistance, which will result in extra cost and hinder application [28,29].

## 4. Conclusions

When it comes to high cycle fatigue tests, costs are often very high, and so is the time period. This paper proposes an accelerated bending fatigue experiment of a crankshaft based on a modified particle filtering algorithm to shorten the experimental process. Select the remaining fatigue life prediction result to substitute for the actual experimental data on analysis of fatigue properties of the part. The main results from this research are the following:

(1) There will inevitably be some prediction errors for the remaining fatigue life of a crankshaft when using the traditional particle filter algorithm, which is mainly due to the effect of low-contribution particles and constant model parameter values. In the modified algorithm, there is an attached corresponding optimization method to improve predictions.

(2) From the comparison between the improved particle filtering algorithm and the traditional particle filtering algorithm, the modified particle filtering algorithm can accurately predict the remaining fatigue life under a shorter experimental time, so that the corresponding saved time is more evident. It is also an advantage on an engineering application basis.

In this paper, the material of the crankshaft is high-strength alloy steel. Additionally, for the crankshaft in modern engineering, another type of commonly used material is nodular cast iron, especially for the gasoline engine. For this kind of cast iron, the crack propagation behavior is obviously affected by the graphite particles distributed within the specimen. Whether the proposed method is still useful in researching the fatigue behavior of the crankshaft made by this kind of material is still unknown. In this way, more comprehensive evaluation of the accelerated bending fatigue experiment can be achieved. In addition, some other techniques such as machine learning can provide high enough accuracy in predicting the fatigue strength of the steel material [30]. Moreover, for the fatigue life, the data usually shows more obvious dispersion property. Thus, more work should be performed in this field to further analyze the fatigue property of the modern crankshaft.

## Figures and Tables

**Table 1:** The main chemical components of the steel 42CrMo.

Component	Percentage/%
C	0.45
Si	0.37
Mn	0.8
S	<0.035
P	<0.035
Cr	1.2
Ni	0.3
Cu	0.3
Mo	0.25

**Table 2:** The fatigue test results of the crankshaft.

Specimen Number	Load Amplitude/N·m	Stress Amplitude/MPa	Frequency/Hz	Fatigue Life/Cycles
1	6017	673.9	41.21	779,762
2	5688	637.1	40.91	3,103,235
3	5352	599.4	40.55	5,511,350
4	5497	615.7	40.71	4,328,128
5	5988	670.7	41.18	868,299
6	6074	680.3	41.26	543,448
7	5207	583.2	40.38	5,624,627
8	6278	703.1	41.43	575,953
9	6133	686.9	41.31	327,416
10	6104	683.6	41.28	402,108

**Table 3:** The prediction results based on different approaches.

	Errors of Different Approaches		Timesaving Percentage	
Specimen Number	Traditional PF	Modified PF	Traditional PF	Modified PF
N2	5.3%	1.2%	11.2%	25.7%
N3	5.1%	1.9%	12.7%	27.7%
N4	16.6%	1.4%	24.2%	48.4%
Mean value	9%	1.5%	16.3%	33.9%

## Data Availability

The original contributions presented in this study are included in the article. Further inquiries can be directed to the corresponding author.
